# Correction to “Mastication Wear of Two Low Profile Attachment Systems for Overdenture: An In Vitro Study”

**DOI:** 10.1155/bmri/9873052

**Published:** 2026-08-11

A.N. Sassi, C. Todaro, G. Isola, et al., "Mastication Wear of Two Low Profile Attachment Systems for Overdenture: An In Vitro Study", BioMed Research International, 2022, 6469890, https://doi.org/10.1155/2022/6469890

In the article, Paola Blasi was missing from the author list. The corrected author list and affiliations are shown below, and have been corrected in the article:

Alessandra Nicole Sassi^1^, Claudia Todaro^1^, Gaetano Isola^2^, Ivano Bortolini^3^, Paola Blasi^4^, Ruggero Rodriguez y Baena^1^,Stefano Storelli^5^, Saturnino Marco Lupi^1^

1. Department of Clinical Surgical, Diagnostic and Pediatric Sciences, University of Pavia, 27100 Pavia, Italy
2. Department of General Surgery and Surgical‐Medical Specialties, University of Catania, Via S. Sofia 78, Catania 95124, Italy
3. Prosthodontic Lab, Via Driovilla 5, 31050 Miane, Italy
4. Private Practice, 16100 Genova, Italy
5. Department of Biomedical, Surgical and Dental Sciences, University of Milan, Milan, Italy

We apologize for this error.

